# Heart Development and Regeneration in Non-mammalian Model Organisms

**DOI:** 10.3389/fcell.2020.595488

**Published:** 2020-10-29

**Authors:** Jianhong Xia, Zhongxuan Meng, Hongyue Ruan, Wenguang Yin, Yiming Xu, Tiejun Zhang

**Affiliations:** ^1^GMU-GIBH Joint School of Life Sciences, Qingyuan People’s Hospital, Guangzhou Medical University, Guangzhou, China; ^2^Guangzhou Regenerative Medicine and Health Guangdong Laboratory, Guangzhou, China; ^3^State Key Laboratory of Respiratory Disease, National Clinical Research Center for Respiratory Disease, Guangzhou Institute of Respiratory Health, The First Affiliated Hospital of Guangzhou Medical University, Guangzhou, China; ^4^School of Basic Medical Sciences, The Sixth Affiliated Hospital of Guangzhou Medical University, Guangzhou Medical University, Guangzhou, China

**Keywords:** heart, development, regeneration, non-mammalian, cardiovascular disease, animal model

## Abstract

Cardiovascular disease is a serious threat to human health and a leading cause of mortality worldwide. Recent years have witnessed exciting progress in the understanding of heart formation and development, enabling cardiac biologists to make significant advance in the field of therapeutic heart regeneration. Most of our understanding of heart development and regeneration, including the genes and signaling pathways, are driven by pioneering works in non-mammalian model organisms, such as fruit fly, fish, frog, and chicken. Compared to mammalian animal models, non-mammalian model organisms have special advantages in high-throughput applications such as disease modeling, drug discovery, and cardiotoxicity screening. Genetically engineered animals of cardiovascular diseases provide valuable tools to investigate the molecular and cellular mechanisms of pathogenesis and to evaluate therapeutic strategies. A large number of congenital heart diseases (CHDs) non-mammalian models have been established and tested for the genes and signaling pathways involved in the diseases. Here, we reviewed the mechanisms of heart development and regeneration revealed by these models, highlighting the advantages of non-mammalian models as tools for cardiac research. The knowledge from these animal models will facilitate therapeutic discoveries and ultimately serve to accelerate translational medicine.

## Introduction

Cardiovascular disease is a common disease that is a serious threat to human health (Waardenberg et al., 2014). According to WHO reports, the number of people died from cardiovascular diseases in 2016 was estimated to be 17.9 million, representing 31% of all global deaths (Ruan et al., 2018). With the growing age of the human population, the decline in heart function has placed a severe burden on the management of human health. Although surgeons have developed many effective procedures to relieve heart disease symptoms and replace heart functions, there are no substitute for the patient’s natural heart (Galdos et al., 2017). Even with the most advanced and well-developed treatment methods, more than 50% of survivors of cardiovascular accidents can’t live completely on their own.

Our understanding of human heart development and cardiac diseases is still inadequate. The molecular and genetic networks of heart diseases are complicated, leading to a variety of structural and functional cardiac phenotypes. The molecular mechanisms, prevention and treatment of various forms of cardiovascular diseases have always been hot topics in biomedical research.

Both the morphogenesis and the underlying genetic networks of heart development are conserved across species (Evans et al., 2010; Staudt and Stainier, 2012). This conservation allows researchers to model human heart developmental disorders in a panel of animal models. The research works in non-vertebrate animals provide a basic framework of the signaling pathways and transcription networks and their control of the spatiotemporal integration of cardiac development, and offer new insights into the general aspects of cardiogenesis and regeneration in vertebrates and humans.

Here, we introduced four popular non-mammalian animal models (fruit fly, zebrafish, frog, and chicken), focusing on their cardiac development, and recent progress in heart regeneration. A deeper understanding of the magical evolution of the heart, and the underlying molecular regulatory networks, will shed light on the essential factors involved in heart development and regeneration, and treatment of cardiac diseases.

## Heart Development Is Highly Conserved Across Species

Heart development is a delicately regulated process and is highly conserved across species. During embryonic development, the heart undergoes a dynamic and complex process starting with the convergence of the myocardial and endocardial precursors to the midline, and culminating in the morphogenesis of these differentiated cells into a complex organ both morphologically and functionally (Sylva et al., 2014). The developing heart of most vertebrates is practically indistinguishable through the linear heart tube stage into the early stages of looping morphogenesis (Warkman and Krieg, 2007).

The genetic networks regulating vertebrate heart development are highly conserved (Staudt and Stainier, 2012). This conservation allows researchers to model human heart developmental disorders in animal models. Across the long evolutionary history, an array of typical animal models in different evolutionary statuses have been screened out and become powerful tools for investigating pathologies of cardiac disease, including mammalian (mouse, pig, and rabbit, etc.) and non-mammalian (fruit fly, frog, zebrafish, and chicken, etc.) models. They both have advantages and disadvantages in the study of heart disease pathology. For example, mice are genetically tractable but are difficult to perform real-time imaging. Zebrafish embryos are excellent for non-invasive live imaging, but their small size and clutch numbers make systematic proteomic analysis difficult. The data and conclusions obtained from different animal models can be mutually confirmed and complement each other, and jointly construct a panoramic view of understanding of heart development and cardiovascular diseases (Afouda and Hoppler, 2009; Bossers et al., 2018).

**FIGURE 1:**
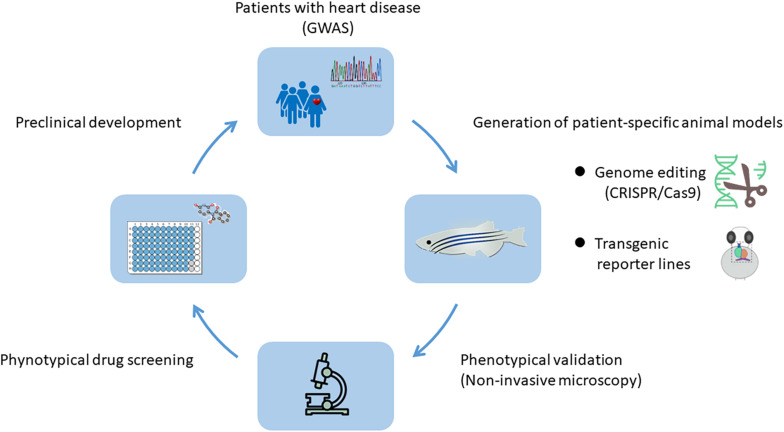
Genome-Wide Association Studies (GWAS) and animal models: a powerful combination for cardiovascular research. With the help of genome editing technology and the genetic information obtained by GWAS from human genetic disease families, animal models with patient-specific heart diseases can be established. The system enables the *in vivo* analysis of underlying pathogenic mechanisms and is highly suitable for high-throughput drug screening, thereby providing a large number of preclinical data.

The heart development in animal models can be effectively studied at the genetic level to reveal genes that control the fate of cardiac cells and determine the response of cardiac cells to signals from neighboring micro-environment. In recent years, with the rapid development of genome editing technology, high-throughput sequencing and single cell sequencing technology, combined with the numerous genetic data of inherited diseases obtained by Genome-Wide Association Studies (GWAS) (Liu et al., 2015; Nikpay et al., 2015), researchers can establish animal models of specific heart diseases recapitulating human conditions and comprehensively study the underlying pathogenic mechanisms (Figure 1).

## Heart Development in Non-Mammalian Model Organisms

In most vertebrate, the heart development can be artificially divided into the following stages: (1) induction of cardiogenic cells from the embryonic mesoderm; (2) coalescing of these cells into a linear heart tube; (3) cardiac looping and asymmetric development; and (4) chamber specialization and growth. These processes are highly similar in most vertebrates, which indicates the evolutionary conservation of the heart building plan.

Due to the evolutionary conservation of development, observations in non-mammalian models have contributed basic insights into the heart developmental mechanisms and allow us to extrapolate to human. Despite the differences in final form and physiology, there are many important discoveries in human heart development guided by studies of non-mammalian models. A well-known example is the identification of the contribution of neural crest cells (NCCs) to outflow tract (OFT) remodeling, which was first identified by the classic fate mapping experiments using chimeric embryos of quail donor and chicken host (Kirby et al., 1983). The involvement of NCCs in OFT development has been further validated in mice and human (Kaartinen et al., 2004; Brown and Baldwin, 2006).

### Fruit Fly (*Drosophila melanogaster*)

Among the four non-mammalian animal models aforementioned, *Drosophila* is the only invertebrate, and has the most distant from human in evolutionary status. Due to evolutionary conservation, from the research works of *Drosophila*, we can understand the most basic building blocks of cardiac development (Ugur et al., 2016; Zhu et al., 2017).

The heart of *Drosophil*a (also known as the dorsal vessel) is a simple tubular structure under the surface of dorsal midline, mainly composed of two kinds of cells, cardiomyocytes inside of the contractile cardiac tube and pericardial cells outside the cardiac tube without contractility (Rotstein and Paululat, 2016). The contraction of cardiomyocytes pumps the hemolymph from the posterior cavity of the heart into the aorta, and then leaves the heart tube to spread into the open body cavity.

In both *Drosophila* and vertebrate embryos, the heart originates from two rows of bilaterally symmetric mesoderm cells. These mesoderm cardiac progenitor cells migrate most distally from the point of invagination during gastrulation, and eventually merge in the middle line to form a heart tube. Subsequent looping and septa formation lead to the multiple-chambered heart in vertebrates while the *Drosophila* heart remains tubular (Bodmer, 1995).

### Zebrafish (*Danio rerio*)

Zebrafish have a single circulation system. The fish heart is two-chambered, with the single atrium and ventricle connected by an atrio-ventricular valve (Beis, 2005). Blood enters the heart through the atrium, then is pumped by the ventricle and ejected into the circulation through the bulbus arteriosus, a prominent outflow tract.

The zebrafish heart development is roughly divided into the following stages: At 5 h post-fertilization (hpf), the cardiac progenitor cells appear in the lateral marginal zone of the blastula. These progenitors migrate toward the midline of the embryo during gastrulation. At about 15 hpf, the cardiac progenitor cells fuse into the cardiac disk with endocardial cells in the center lined by ventricular and atrial myocytes. At 22 hpf, the heart begins regular contraction. From 24 to 28 hpf, the cardiac disk elongates into a linear heart tube and begins leftward migration. The cardiac valve and the two chambered heart has formed by 48 hpf (Sarmah and Marrs, 2016; Gut et al., 2017).

Unique characteristics of zebrafish make them particularly attractive for cardiovascular research. Its advantages include external fertilization, small size, early optical transparency, easy genetic manipulation, and the ability to survive without a functional cardiovascular system at early stages of development (Poss, 2002). It’s easy to carry out forward or reverse genetics manipulations in zebrafish embryos (Grant et al., 2017). There is a large number of gene-modified zebrafish lines, including mutant lines and transgenic lines with various tissues, organs or specific cells labeled with fluorescent proteins (Verma and Parnaik, 2017). Advances in microscopy and non-invasive imaging have utilized the transparency of zebrafish embryos to monitor the dynamic cellular events that transpire during cardiac morphogenesis. Zebrafish embryos have also been used as a high-throughput drug discovery platform (Zhu et al., 2018). The embryos are small in size, allowing most of the chemical compounds to be directly diluted into the water and readily diffuse into embryos. The combination of these salient features makes zebrafish a high-throughput but low-cost model organism that combines the advantages of forward and reverse genetics with phenotype-driven drug screenings (MacRae, 2010; Kithcart and MacRae, 2017).

### Frog (*Xenopus laevis*)

*Xenopus* has a three-chamber heart that consists of two atria and one ventricle. The right side of the heart receives deoxygenated blood from the body, and the left side receives freshly oxygenated blood from the lungs. The two streams of blood mix together in the ventricle, emitting a concoction that is not completely oxygenated to the rest of the body (Afouda and Hoppler, 2009; Hempel and Kühl, 2016; MacColl Garfinkel and Khokha, 2017).

The heart of *Xenopus* starts out as two bilateral patches of the specified mesoderm on the dorsal side of the embryo at the onset of gastrulation. Gastrulation movements make the heart patches move dorso-anteriorly. During neurulation, the cardiac cells move as two distinct populations on either side of the embryo toward the forward end of the mesoderm sheet as it engulfs the yolk and encompasses the embryo (Harvey, 2002). At the ventral midline the two heart patches fuse to form a simple linear tube. As development proceeds, the cardiac tube begins to undergo looping. The atria then undergoes a slow septation process, leading to the formation of a three-chambered heart (Kaltenbrun et al., 2011; Hempel and Kühl, 2016). In contrast to zebrafish, the separated atria in the *Xenopus* heart allows for the study of atrial septal defects.

*Xenopus* is already a well-established model for studying development and regeneration. *Xenopus* embryos are large and robust, exhibiting a remarkable ability to heal after microsurgery. These features make it relatively easy to perform powerful “cut and paste” transplant experiments to study tissue interactions, especially in heart development. Such methods can be used to separate cardiogenic mesoderm from non-cardiogenic mesoderm, to study the progress of cardiac fate decision and the function of inducers/repressors of heart development (Nenni et al., 2019).

### Chicken (*Gallus gallus*)

Birds and mammals have a fully separated ventricle and a bona fide four-chambered heart. The four-chambered heart ensures the formation of a high-pressure systemic circulation and a low-pressure pulmonary circulation, allowing for high metabolic rates and maintenance of body temperature.

Embryonic chicken is a commonly used vertebrate model with the advantage of resembling human heart structure with four-chamber/four-valve configuration and enabling clinically relevant surgical manipulations. It resembles the human heart anatomy more closely than other non-mammalian model organisms (Wittig and Münsterberg, 2016; Lansford and Rugonyi, 2020). Due to the long generation time, genetic methods are not straightforward in chickens. However, *in vivo* accessibility allows the use of transient gain- and loss-of-function approaches to compensate for this deficiency.

## Molecular Mechanisms of Heart Development in Non-Mammalian Model Organisms

With the development and advancement of technologies for assessing the genomes, transcriptomes, and proteomes under different conditions, considerable molecular data on heart development and diseases have been collected from non-mammalian models. Starting from simple non-mammalian models, such as *Drosophila* with a heart composed of only about 80 mature cardiomyocytes (Rotstein and Paululat, 2016), some conserved molecular mechanisms have been discovered (Alvarez, 2003; Ahmad, 2017). Subsequently, homologous genes and pathways can be further investigated in higher model organisms. One recent example is the role of the CCR4-NOT in cardiovascular development. CCR4-NOT is a conserved multi-protein complex regulating gene expression, mRNA stability, and mRNA turnover. In a reverse-genetic screen for genes affecting adult heart function under stress conditions in *Drosophila*, several members of the CCR4-NOT complex were identified to cause dilated cardiomyopathy (Neely et al., 2010). Further molecular mechanism studies revealed that CCR4-NOTcomplex is a global regulator of cardiac gene expression, which links metabolism of RNA and epigenetic gene regulation and plays crucial roles in coordinating functional gene networks in hearts. The function was subsequently verified in other higher animal models, such as mice (Yamaguchi et al., 2018) as well as humans (Zhou et al., 2017).

The *Drosophila* heart is one of the earliest systems in which the key mechanisms regulating cardiac development have been investigated at the genetic and molecular level. *Drosophila* has been widely used to decipher the affected molecular and cellular pathways in CHDs and to identify their genetic modifiers. Several genes that cause human CHDs are also present in *Drosophila*. Their roles in heart development, maintenance, or physiology are the same or similar to their counterparts in humans (Qian et al., 2011; Zhu et al., 2017). The identification of highly conserved transcription factors that play crucial roles in cardiac development in *Drosophila* led to the identification of a conserved cardiogenic network in vertebrate. These transcription factors include *Tinman* (Nkx2.5) (Bodmer, 1993), *pannier* (GATA4) (Lovato et al., 2015), *neuromancer* (Tbx20) (Qian et al., 2005, 2008), and HAND (HAND2) (Han, 2006; Lo et al., 2007; Hallier et al., 2015).

Through genetic screening strategies, novel genes required for cardiovascular development have also been identified in zebrafish, *Xenopus*, and chicken models, which contribute to advance the discovery of heart development mechanisms. For example, *faust* (*fau*^*tm236a*^) is an ENU-induced, embryonic lethal recessive mutation in zebrafish that selectively disrupts formation of the definitive heart tube (Chen et al., 1996). Positional cloning identified that the *fau* locus encodes the zinc finger transcription factor Gata5. Further mechanism analysis provides the first *in vivo* evidence that Gata5 is required for the production of myocardial precursors and the expression of several myocardial genes including nkx2.5 (Reiter et al., 1999). Experiments in chicken (Ban et al., 2013) and *Xenopus* (Jiang and Evans, 1996; Haworth et al., 2008) provide further evidence for the role of Gata factors in cardiovascular development.

Heart development and function was controlled by an evolutionary conserved orchestra of transcription factors and signaling pathways (Schlesinger et al., 2011). Recently, the role of epigenetic and post-transcriptional mechanisms (such as histone modification and microRNA) have been identified (Alfar et al., 2018).

The cells that make up the heart originate from the cardiac mesoderm during early embryonic development. The expression of basic-helix-loop-helix (bHLH) transcription factor Mesp1 (Mesoderm Posterior 1) is considered to be one of the earliest markers of cardiac precursors (Saga, 2000). As a master regulatory factor, Mesp1 induces a panel of down-stream genes (HAND2, GATA4, and Nkx2.5, etc.), which facilitate the specification of cardiac progenitors and formation of the heart field (Bondue and Blanpain, 2010).

The different anatomical structures of the heart come from two groups of cardiac progenitor cells with distinct molecular characteristics, referred to as the first heart field (FHF) and the second heart field (SHF). The cells in the FHF mainly differentiate to form the left ventricle and parts of the atria. Adjacent to the FHF, the SHF contributes predominantly to the right ventricle, atria, inflow and outflow tracts. Compared to FHF, SHF has stronger cell proliferation ability with slower differentiation. SHF cells are multi-potent that can be differentiated into various cardiac cell types, such as cardiomyocytes, smooth muscle cells, endothelial cells, and fibroblast cells, while FHF cells mostly generate cardiomyocytes. FHF cells express Tbx5, HCN4, and Nkx2.5. SHF cells specifically express transcription factors Islet1, Tbx1, Six2 and growth factors Fgf8/10 (Poelmann and Gittenberger-de Groot, 2019). The expression pattern of these genes in FHF and SHF cells determine their differentiation time points.

During embryonic development of all vertebrates, the looping of heart tube leads to the conversion of its anterior-posterior polarity into left-right polarity. The direction of heart looping depends on the left-right asymmetry established through the asymmetric expression of the genes Nodal, Lefty and Pitx2 on the left side of the body (Dykes, 2014), which is controlled by Notch, Hedgehog, Wnt, and BMP signaling pathways.

As a new class of regulators of cardiomyogenesis and heart diseases, microRNAs have been proved to be essential for cardiac development and indispensable for maintaining the homeostasis and function of the heart. The overall disturbance of the cardiac miRNAome by the deletion of the microRNA processing enzyme Dicer leads to dilated cardiomyopathy (Chen et al., 2008). The expression of cardiac transcription factors is also regulated by several microRNAs. For example, HAND2 was identified as one of the miR-1 targets. miR-1 also targets histone acetylase 4 (HDAC4), and HDAC4 inhibits cardiomyogenesis by down-regulation of GATA4 and Nkx2.5 expression (Zhao et al., 2005; Thum et al., 2008).

## Heart Regeneration in Non-Mammalian Organisms and Applications in Regenerative Medicine

In humans, myocardial infarction is a common cause of cardiac injury. Unfortunately, the human cardiac cells show very limited ability for regeneration to replace the lost cardiomyocytes. The necrotic cardiomyocytes are gradually replaced with fibrotic tissue through a re-modeling process, which can eventually lead to heart failure (Nadal-Ginard et al., 2003; Chiong et al., 2011; Frangogiannis, 2015). Regenerative methods to replenish the cardiomyocytes should be of great potential to resolve this problem.

Research of cardiac regeneration focuses on the development of a wide spectrum of model organisms to decipher the mechanisms and factors involved in heart repair. For decades, animal models of cardiomyocyte regeneration have been restricted to non-mammalian organism models (Uygur and Lee, 2016; Vivien et al., 2016), such as teleost fish and amphibians.

Zebrafish possess highly efficient cardiac regenerative capacity throughout their lifetime, providing novel insights into the understanding of human cardiac regeneration (Bournele and Beis, 2016; Bossers et al., 2018). The zebrafish heart can reform a fully functional heart even after substantial damages (Sehring et al., 2016; Tahara et al., 2016; González-Rosa et al., 2018).

*Xenopus* is regarded as a leading model of regeneration research. A new study using *Xenopus laevis* as a model of heart regeneration showed that the tadpole hearts can regenerate until the larvae reach metamorphosis. Excessive or reduced thyroid hormones reduce the cardiac regenerative capacity, suggesting that fine-tuning the availability of thyroid hormones may be necessary for heart regeneration (Marshall et al., 2019).

Interestingly, even animal species with close evolutionary kinship may have very different heart regeneration capabilities. In cyprinids, while the hearts of zebrafish and goldfish (Grivas et al., 2014) have strong regenerative ability, the medaka (*Oryzias latipies*) heart can be scarred after injury without regeneration (Ito et al., 2014). Young adult *Xenopus tropicalis* can regenerate heart tissue after ventricular resection, while mature adults of the closely related species *Xenopus laevis* are not capable of cardiac regeneration (Liao et al., 2017, 2018). Exploring the differential responses of these closely related species to cardiac injury will provide a unique opportunity to identify new factors and inform new therapeutic approaches for cardiac regeneration.

## Concluding Remarks

Over the past few decades, there is a growing understanding of heart development and regeneration in the non-mammalian model organisms, which has provided a valuable reference for related research in mammalians. A deep understanding of the formation and development of the heart has allowed cardiac biologists to significantly intervene in the challenge of therapeutic heart regeneration. In non-mammalian animal models, various platforms can be established quickly, including forward and reverse genetic manipulations and high-throughput omics studies. The latest gene editing tools, including CRISPR/Cas9 and high-precision base editors, are continuously being optimized for the use in non-mammalian animals. Massive genetic data brought by the rapid development of big data era, in combination with new genetic tools, deep sequencing, as well as further advances in microscopy imaging becoming available, there is no doubt that non-mammalian animal models will continue to reveal novel insights into cardiac development and contribute to cardiac repair. Deciphering the cellular and molecular mechanisms that regulate cardiac development and regeneration will promote the development of therapeutic strategies to cure heart diseases, impacting millions of people worldwide.

## Author Contributions

JX wrote the initial draft of the manuscript. ZM and HR wrote the manuscript. YX and WY contributed to design, writing, and final approval of the manuscript. TZ contributed to conception and design, financial support, administrative support, writing, and final approval of the manuscript. All authors contributed to the article and approved the submitted version.

## Conflict of Interest

The authors declare that the research was conducted in the absence of any commercial or financial relationships that could be construed as a potential conflict of interest.
